# Garrison Institute on Aging—Lubbock Retired and Senior Volunteer Program (RSVP) Provides Services to South Plains, Texas

**DOI:** 10.3389/fnagi.2015.00215

**Published:** 2015-12-08

**Authors:** Joan Blackmon, Annette N. Boles, P. Hemachandra Reddy

**Affiliations:** ^1^Garrison Institute on Aging, Texas Tech University Health Sciences CenterLubbock, TX, USA; ^2^Cell Biology and Biochemistry, Texas Tech University Health Sciences CenterLubbock, TX, USA; ^3^Neuroscience/Pharmacology, Texas Tech University Health Sciences CenterLubbock, TX, USA; ^4^Neurology, Texas Tech University Health Sciences CenterLubbock, TX, USA

**Keywords:** Retired Senior Volunteer, education, Alzheimer's, aging, community service

## Abstract

The Texas Tech University Health Sciences (TTUHSC) Garrison Institute on Aging (GIA) was established to promote healthy aging through cutting edge research on Alzheimer's disease (AD) and other diseases of aging, through innovative educational and community outreach opportunities for students, clinicians, researchers, health care providers, and the public. The GIA sponsors the Lubbock Retired and Senior Volunteer Program (RSVP). According to RSVP Operations Handbook, RSVP is one of the largest volunteer efforts in the nation. Through this program, volunteer skills and talents can be matched to assist with community needs. It is a federally funded program under the guidance of the Corporation for National and Community Service (CNCS) and Senior Corps (SC). Volunteers that participate in RSVP provide service in the following areas: food security, environmental awareness building and education, community need-based volunteer programs, and veteran services.

## Retired and senior volunteer program

Since 1997, Texas Tech University Health Sciences Center (TTUHSC) has considered aging one of its highest priorities. As a result, the Board of Regents established the Garrison Institute on Aging (GIA) in 2000 with a mission to promote healthy aging, conduct cutting-edge research and provide innovative educational experiences to health care professionals and the public (Reddy et al., 2015). Because aging is a key focus for TTUHSC, the sponsorship of Lubbock RSVP not only furthers its mission to serve the community (Corporation for National and Community Service, 2008) but it also aids in advancing the mission of the GIA.

TTUHSC GIA's unique organizational structure provides educational and community outreach opportunities offered to students, health care professionals, and the public. One of these community outreach opportunities is the Lubbock Retired and Senior Volunteer Program (RSVP). RSVP, the largest volunteer network in the nation for adults age 55 and over, links volunteers and their skills and talents to identified for community needs. Lubbock RSVP volunteers do not receive financial incentives or stipends (Corporation for National and Community Service, 2008). However, the Lubbock RSVP project does provide the volunteers with limited liability insurance. Volunteers in the Lubbock RSVP program determine how many hours a week they can assist and establish their own volunteering schedule. According to the website—www.nationalservice.gov, the Corporation for National and Community Service (CNCS) is a federal agency that funds RSVP and helps more than 5 million Americans improve the lives of their fellow citizens through service (Corporation for National and Community Service, 2007). The National and Community Service Trust Act established in 1993. CNCS serves many purposes, one which includes to build on existing organizational service infrastructure of Federal, state, and local programs and agencies to expand full-time and part-time service opportunities for all citizens (Corporation for National and Community Service, 2008). Through, the support and resources of CNCS, more 500 volunteers participate in the Lubbock RSVP program and provide ~105,000 h of services on a yearly basis. The differential benefits of volunteering have substantiated the long-term impact on seniors. Research findings indicate that older volunteers experience increased life satisfaction and more social contact as well as a perceived improvement in health when compared with younger volunteers (Van Willigen, 2000). While volunteers serve the community, their service is also beneficial to their health and perceived well-being (Corporation for National and Community Service, 2008). This is noted in research studies by Okun and Shultz that validate the importance of volunteer service in promoting healthy aging among older adults (Okun and Shultz, 2003). Elders who choose to volunteer in the community as a continuation of successful careers not only provide services valued at more than $161 billion in the United States (over 2.4 million annually in Lubbock community), but also improve their chances of living longer and healthier lives.

The mission of Lubbock RSVP is a dedication to addressing community needs by matching the interests, experience, and talents of individuals age 55 and older with rewarding volunteer opportunities. Lubbock RSVP is essentially a bridge between non-profit agencies (with community priorities) and services for the Lubbock County community. The non-profit agencies that participate in the RSVP program are called stations. According to the CNCS website, “A volunteer station is a public agency, secular or faith-based private non-profit organization or proprietary health care organization that accepts the responsibility for assignment and supervision of RSVP volunteers (Corporation for National and Community Service, 2008). These stations are designated community priorities in need of assistance from volunteers. Volunteer stations can include multi-purpose centers, home care agencies or other areas of services, and each volunteer station must be licensed or otherwise certified, when required by appropriate state or local government. Private homes are not volunteer stations (Corporation for National and Community Service, 2008). The 500 volunteers that participate in the Lubbock RSVP program are matched to stations based on previous work experience, talents and skills. Examples of the stations are: Meals on Wheels, Veterans Clinic, Friends of Library and South Plains Food Bank (See Appendix in Supplementary Material for a full list of the stations.). Areas served include information and guidance on alleviating food insecurity, environmental and healthcare programs for low-income families, non-medical health-related information, and care in the following areas: environmental awareness-building and education, community-based volunteer programs, and other needs of the public. Lubbock RSVP conducts surveys and questionnaires targeted at the public it serves in order to measure the impact of its programs. Lubbock RSVP aims to help seniors by connecting them with volunteer opportunities at stations that increase their knowledge of water and resource conservancy through classes, provide sources for household assistance to low-income families, provide non-profit resources benefiting those in need, and decrease hunger through Lubbock RSVP volunteers who deliver meals to home-bound citizens and various food pantries.

The Lubbock RSVP is led by Advisory Council that is guided through by-laws that identify Lubbock RSVP authority, purpose, and scope; and that define its Advisory Council membership terms and eligibility. The Advisory Council currently has 13 members who assist in guidance in its mission, goals and objectives of the program. The council not only provides feedback to the Director on a monthly basis, but also volunteers for assistance at special RSVP events.

The Lubbock RSVP project will consistently strive to meet the needs of both a constantly changing diverse society and the other senior community that are served. As a result, the program conducts evaluations in order to determine the health of the program and identify areas for improvement.

## Volunteer recruitment and development

Under the auspices of the GIA, Lubbock RSVP pursues dedicated volunteers who comprise a diverse population in order for the community to identify more readily with the volunteers and the Lubbock RSVP programs. The Lubbock RSVP office seeks volunteers interested in filling positions that will allow them to develop and build a wide range of skills—from home food delivery to respite care to educational services—they can pass the legacy onto persons in the community. In order to recruit new members, Lubbock RSVP publishes a monthly article in the *Golden Gazette*, a local senior and community newspaper, places flyers in volunteer stations and promotes the volunteer opportunities in the RSVP quarterly newsletter. The annual spring forum and movie night are also utilized for recruitment. Interested persons contact Lubbock RSVP for information and a volunteer enrollment form. Volunteers are matched on their previous their work experience, skills, and hobbies.

Upon completion of enrollment forms, Lubbock RSVP staff enters this information into the volunteer database where it can be sorted to obtain information to match volunteers with available volunteer positions at stations. The RSVP staff strives to ensure that each volunteer's skills and desires match with needs of each particular station. Station volunteer coordinators provide additional training, screening and orientation. Persons who indicate on the enrollment form that their life experiences include leadership roles are encouraged to volunteer for positions that strengthen and assist Lubbock RSVP stations that focus on capacity building. Programs that include capacity building include: the Coalition of Community Assistance Volunteers (tax assistance), Senior Corps of Retired Executives (S.C.O.R.E.), and the South Plains Association of Governments (ombudsmen). Volunteers are encouraged to contact and work with Lubbock RSVP staff and station volunteer coordinators for any training or technical assistance required in order to perform assignments.

## Lubbock RSVP volunteer activities

The activities that Lubbock RSVP volunteers are engaged in are based on the community's needs. In and around the Lubbock area, about 730 people are living in some form of homelessness, either in homeless shelters, “doubled up” with other families, or on the streets (Texas Homeless Network, 2013). Families comprise more than one third of the homeless. According to the 2010 census, 20% (56,045) of the residents of Lubbock County are living at or below the poverty level, and 14% (20,130) between the ages of 21 and 64 are disabled (United States Census Bureau)^1^. Families with such limited resources, and/or with fixed incomes, or are unemployed, homeless or disabled, are required to make very difficult choices regarding daily expenses and the basic necessities of life. Research by the South Plains Food Bank found that about 55,000 people in Lubbock County are receiving food assistance each year. Fifty-seven percent of their clients report having to choose between paying for food and paying for utilities. The number of hungry people on the streets of Lubbock continues to increase. One in four children and one in five adults in Lubbock are at risk of food insecurity, defined as “the limited and uncertain availability of nutritional, adequate, and safe food or the inability to acquire acceptable foods” (Manna, 2013)^2^.

With this urgent need, Lubbock RSVP volunteers assist the South Plains Food Bank. The South Plains Food Bank has several programs in which volunteers can become involved. Kids Café is an after-school feeding program designed to combat childhood hunger. Volunteers assist with food preparation and service, and their talents are also used in tutoring children. A second program is the 15-acre apple orchard that produces over 25,000 pounds of apples—fresh fruit for the needy. Volunteers can work on a 5.5-acre farm where vegetable poundage produced is about 100,000 pounds annually. Another program is GRUB (Growing Recruits for Urban Business), a program where volunteers teach teens and young adults about life and job skills they can acquire on farms and community gardens. Lubbock RSVP volunteers are trained to assist with unloading deliveries, stocking shelves, registering and servicing clients, packaging and preparing food boxes, filling backpacks for children, participating in food drives, collecting food vouchers, and collecting and reporting client data.

Lubbock RSVP also collaborates with a non-profit agency, Lubbock Meals on Wheels (LMOW) the mission of which is to eliminate hunger and waste of food resources; to give hope to the hungry; to provide food to people who are homebound, elderly or disabled; and to means whereby people in need can access and/or acquire food. The LMOW provides and delivers over 700 nutritious, home delivered meals to those who are homebound, elderly or disabled. It also seeks to break the social isolation experienced by the homebound by providing warm, caring, friendly contact through volunteers and enabling them to live independently. In addition, 200 weekend packs are prepared and delivered for clients who do not have access to a healthy nutritious meal on weekends (News on Wheels, 2015). There continuous to be a wait list for recipients and LMOW is continually researching avenues to increase this service. New volunteers are trained by Lubbock Meals on Wheels to assist with meal preparation and meal delivery.

Another critical area where volunteers are needed is in thrift stores and in constructing clothing and bedding that provide comfort and warmth. The need for warm and usable clothing and bedding is a serious issue for income-challenged residents who are on fixed incomes, are unemployed, or are homeless. Two agencies have been identified to meet community needs: Catholic Family Services Resale Shop and St. Paul's Thrift House. These thrift stores do not have the capacity to meet this challenge within their own staff. As a result, Lubbock RSVP volunteers assist these organizations to wash, clean, price, sort, tag, display, repair, and remodel, sanitize, and compassionately interact with their clients. In addition, these agencies provide emergency funding for medical, housing and personal needs that are urgent and necessary for clients.

For nursing homes to care for all of the non-medical, comfort needs of the elderly population in the Lubbock area is cost-prohibitive, and they do not have the staff resources to meet this challenge either. Through, the Lubbock RSVP Comfort Corps, Lubbock RSVP volunteers provide to the residents of nursing homes a variety of hand-made items, including lap blankets. Additional items are also provided for agencies that serve the public. Some examples of these are Ronald McDonald Charities, Hope Lodge (American Cancer Society), My Father's House, as well as local hospitals and Veterans Clinics.

Between 2004 and 2008, there were 149 cases of breast cancer diagnosed in Lubbock County. The American Cancer Society (ACS) works with many breast cancer patients in the Lubbock community through the Reach for Recovery Program. Over the past year, ACS helped over 100 breast cancer patients who had single, and in some instances, double mastectomies. ACS also finds providing services to all of these patients cost-prohibitive and staff-prohibitive. Through, the Lubbock RSVP Comfort Corps, Lubbock RSVP volunteers construct heart-shaped pillows, tubing pockets and caps for breast cancer patients, to assist in treating and well-being.

Lubbock RSVP Comfort Corps also makes baby shirts, caps, sweaters, booties, and blankets for agencies that help pregnant teens and pre-mature infants. Comfort Corps construct 18-inch blankets to cover incubators in NICU; and caps, scarves, and afghans for food agencies, such as food banks. Comfort Corps also constructs heart pillows and teddy bears for hospitalized children, Buckner's Children Home, and for police and emergency personnel to provide to displaced children.

Lubbock RSVP volunteers spend time cultivating and maintaining the grounds of hospitals, community arboretums, and community museums. They plant trees, hoe gardens, instruct classes, and make presentations to interested persons of all ages in the community, about how to conserve natural resources and to live a “greener” style of life.

Lubbock RSVP provides a valuable asset for the Lubbock community. Each day, thousands of US citizens retire and are looking for fulfillment in their retirement years. Without their valuable skills and work ethic many non-profits would not be able to fulfill their mission and service to the community. Many citizens would go without critical and vital service.

## Program management

The system of managing the Lubbock RSVP day-to-day activities and data is the *Volunteer Reporter* software. This software enables the Lubbock RSVP Program Director and advisory board members to collect, track, analyze, and compare volunteer and donor information, and provides data for program reporting that is required by funding agencies.

Lubbock RSVP's existing and new community partners and volunteer stations are required to sign a “Memorandums of Understanding (MOU)” with the Garrison Institute on Aging. As a community partner and station of RSVP, each entity determines what method will be utilized to report volunteer hours and any changes in the program. MOUs are valid for 3 years and can be amended at any time (see Appendix in Supplementary Materials for a copy of MOU). As a station with RSVP, each group provides information on financial status of entity, ADA accessible, any cost reimbursements that are provided to volunteers (meals or mileage). Reports are generated on *Volunteer Reporter* to review renewal dates of MOUs. The Lubbock RSVP Director provides input to the Volunteer Coordinator and/or Director at each station serving as a performance measure and explains the impact to the community.

## Special events hosted by Lubbock RSVP

The special events hosted by Lubbock RSVP are used to recognize the volunteers and to recruit new volunteers. Lubbock RSVP Recognition Dinner is one of the high points of the year for its volunteers. The Lubbock RSVP Director and Advisory Council recognize the importance of acknowledging the thousands of hours of service donated by RSVP volunteers (Yoshioka et al., 2007). It is important to thank them for making all Lubbock RSVP programs a vital part of the Lubbock community. The recognition dinner serves as an important socialization and retention tool in that volunteers eagerly anticipate the opportunity to meet with other Lubbock RSVP volunteers and friends, and enjoy food and fellowship. Volunteers are provided small mementos of their service. Lifetime Achievement Awards are given to volunteers who have given 4000 (or more) hours of service while members of RSVP. The award includes a letter and certificate signed by the sitting President of the United States and a pin with the Seal of the United States.

Another annual recruitment and retention activity is the Lubbock RSVP Spring Forum. This educational forum is held annually. It is a day-long event where information and presentations are shared to gain knowledge on services available throughout the community, changes in legislature that is important to senior population, important legal documents for the senior population and city services for citizens. The Program Director and Advisory Council serve as hosts for the event. Attendance in prior years includes 200 attendees and 30 local organizations that host vendor booths. Vendors support this event by assisting in providing a light breakfast, lunch and door prizes.

Movie Night is another recruitment, retention and thank you event. It is held in the summer. A classic 1940s movie is viewed while attendees enjoy a memorable hot dog meal with all the trimmings. The event is free and open to members and guests. The activity provides attendees with an opportunity to socialize with other volunteers. Volunteers are encouraged to bring friends who are interested in learning more about RSVP and the volunteer opportunities. 200 volunteers and guests attended the Movie Night in 2014.

In summary, the Lubbock RSVP program continues to make a substantial impact in the Lubbock Community. According to Bureau of Labor Statistics, older adults are more likely to volunteer than their younger counterparts, especially for religious organizations (Economic News Release, 2015). CNCS states that in 2010 volunteers served 8.1 billion hours and estimated a total value of service of $173 million dollars (Corporation for National and Community Service, 2011). Based on the research that exists and the continued success of the Lubbock RSVP program, both the stations and the volunteers are benefitting from the opportunities to volunteer.

## Funding

The Garrison Institute on Aging research and outreach programs are supported by the Garrison Family Foundation, NIH grants AG042178, AG047812, the Texas Department of State Health Services, and Corporation for National Community Service.

### Conflict of interest statement

The authors declare that the research was conducted in the absence of any commercial or financial relationships that could be construed as a potential conflict of interest.

## Footnotes

^1^United States Census Bureau, State and County Quick Facts – http://quickfacts.census.gov.

^2^Manna Quarterly publication of South Plans Food Bank, Inc., Fall 2013 SPFB, Quick Reference Information Sheet, Feb 2013.
