# Supplementary material for: Garrison Institute on Aging—Lubbock Retired and Senior Volunteer Program (RSVP) Provides Services to South Plains, Texas
Source: Front Aging Neurosci. 2015 Dec 8;7:215. doi: 10.3389/fnagi.2015.00215 (PMC4672082; doi:10.3389/fnagi.2015.00215)
Supplement: Supplementary file 1 [file DataSheet1.DOCX]

| **Appendix:**  **Station Name** |
| --- |
|  |
| 19th Street Senior Center |
| American Cancer Society |
| American Wind Power Center |
| Bayer Museum of Agriculture |
| Carillon, Inc. |
| Catholic Charities, Diocese of Lbk. |
| Covenant Med Cntr - Lakeside |
| Covenant Medical Center |
| Exodus Prison Ministry |
| Friends Of The Library |
| Gentiva Hospice |
| Grace Medical Center |
| Health Sciences Center |
| Homestead/Silver Village |
| Hospice Of Lubbock |
| Interim Healthcare Hospice |
| KTTZ-TV/FM |
| Lubbock Meals On Wheels |
| Lubbock Mem Arboretum Fndn |
| Lubbock Sheriff's Department |
| National Ranching Heritage Center |
| Ronald McDonald Charities |
| RSVP Social Services |
| Second Helpings at FUMC |
| South Plains Food Bank |
| South Plains Wildlife Rehabilitation Cnt |
| Sp Genealogical Society |
| Spag-Aging Department |
| St John's United Meth Church/Benevolence |
| St. Paul's Thrift House |
| UMC Activities Center Line Dancers |
| UMC Activities Center Line Dancers-High Class Ladies |
| UMC Activities Center |
| Va Outpatient Clinic |
| Word of Hope Ministry |

**M E M O R A N D U M O F U N D E R S T A N D I N G**

**BETWEEN**

**Lubbock RSVP**

Texas Tech University Health Sciences Center/Garrison Institute on Aging

6630 S. Quaker Ave., Suite E, Lubbock, TX 79413

Phone: 806.743.7787 Fax: 806.743.7816 E-mail: [rsvp@ttuhsc.edu](mailto:rsvp@ttuhsc.edu)

**AND**

**VOLUNTEER STATION_______________________ ___________________________**

Address__________________________________________________________________

Phone#___________________________________________________________________ Fax#_____________________________________________________________________

E-mail address (optional):____________________________________________________

Website:__________________________________________________________________

Name and title of person who will be responsible for instruction and supervision of RSVP Volunteers:

____________________ ­­­­____________________________________________

**This Memorandum of Understanding (MOU) contains basic provisions, which will guide the working relationship between both parties. It may also include a Programming for Impact Addendum. This MOU may be amended, in writing, at any time with concurrence of both parties and must be renegotiated at least every three years.**

**I. THE LUBBOCK RSVP PROGRAM WILL:**

A. Provide orientation about RSVP for the Volunteer Station’s representative.

B. Recruit and interview RSVP Volunteers before referring them to the Volunteer Station.

C. Participate in interviews of RSVP Volunteers with the Volunteer Station’s representative before placement, if desired by either party.

D. Review assignments to determine acceptability for RSVP Volunteers.

E. Mail the *Hours Collection form* to the Volunteer Station’s designated agent on a monthly basis.

**II. THE VOLUNTEER STATION WILL:**

A. Provide a list of planned volunteer assignments to the Lubbock RSVP office. This document will be placed in the folder in which this MOU is filed in the RSVP office.

B. Interview and make final approval or disapproval on assignment of volunteers.

C. Provide orientation concerning the Volunteer Station and the individual volunteer assignments.

D. Provide supervision of RSVP Volunteers on assignment.

E. Provide for adequate safety of volunteers.

F. Investigate and report any accidents and injuries involving RSVP volunteers immediately to the RSVP office. All reports will be submitted in writing.

G. Perform any background, criminal or reference checks on potential volunteers referred by RSVP, as required or deemed necessary and prudent by this station.

H. Supply statistical data on volunteer impact on community needs to Lubbock RSVP. In support of RSVP’s reporting requirements for their funding agency, the Corporation for National and Community Service, the Volunteer Station will assist the RSVP staff in Programming for Impact (PFI) planning and reporting initiatives, providing data or supporting information whenever possible. If the station is under a PFI statement, the statement will be discussed with the Volunteer Station Coordinator and attached to the station’s copy of this MOU.

I. Assist with the Volunteer Hours Collection form as much as possible by (please check):

____1. Returning the monthly Volunteer Hours Collection Form with the Volunteer Coordinator’s signature, or

____2. Instructing volunteers to submit their hours individually to the RSVP office either on the phone or with the calendars provided in the RSVP newsletter.

**Station Type:**

Certification that Station/Agency/Organization is the following type: (Please Circle)

Faith Based Private Non-Profit Public Agency Proprietary Healthcare

If you are a non-profit – please provide documentation providing status information.

**III. MEALS** (please check those that apply)

___A. The Volunteer Station will provide the RSVP Volunteer with meals occurring during hours of volunteer service.

___B. The Volunteer Station will partially participate in the provision of such meals as follows:_______________________________________________________________________

___C. The RSVP Volunteer will provide own meals.

___D. Other:_________________________________________________________________

**IV. OTHER OUT-OF-POCKET EXPENSES –** These will be reimbursed by the Volunteer Station as follows:________________________________________________________________________

**V. GENERAL INFORMATION:**

A. **INSURANCE COVERAGE**: Personal Accident Insurance and Personal Liability Insurance will be provided to the RSVP Volunteer by the RSVP Program.

B. **SEPARATION FROM VOLUNTEER SERVICE**: The Volunteer Station, RSVP Volunteer or RSVP may request separation of a Volunteer from a particular assignment. Discussion of individual separation may occur between RSVP staff, Volunteer Station staff and the RSVP Volunteer to clarify the reasons or resolve conflicts.

C. **CONSULTATION AND EVALUATION:** Volunteer Stations and RSVP staff may confer in order to assess the progress, success and needs of the program.

D. **RELIGIOUS AND POLITICAL ACTIVITIES**: The Volunteer Station will not assign nor ask RSVP Volunteers to conduct or engage in religious, sectarian, or political activity or instruction.

E. **DISPLACEMENT OF EMPLOYEES:** The Volunteer Station will not assign RSVP Volunteers in any position which would displace employed workers.

F**. PROHIBITION OF DISCRIMINATION**: The Volunteer Station will comply with provisions of TITLE VI of the CIVIL RIGHTS ACT OF 1964 and will not discriminate against RSVP volunteers or in the operation of its program on the basis of race, color, national origin, sex, age, political affiliation, religion or on the basis of disability, if the volunteer is a qualified individual with a disability.

G. **ACCESSIBILITY AND REASONABLE ACCOMMODATION:** The Volunteer Station will maintain the programs and activities to which RSVP volunteers are assigned accessible to persons with disabilities and provide reasonable accommodation to allow persons with disabilities to participate in programs and activities. Although we know that not every site can always be totally accessible, we need to insure that our program, considered as a whole, will not exclude anyone because of a handicap.

Is your building handicapped accessible? ____yes ____no

Do you have accessible parking? ____yes ____no

H. **AMENDMENTS**: This Memorandum of Understanding may be amended in writing by concurrence of the Volunteer Station and RSVP.

Volunteer Station Coordinator: ______________________________________ Date: ______________

Title:___________________________________________________________

The Volunteer Station representative will serve as liaison with RSVP and will be responsible for volunteer orientation and supervision.

Signature of RSVP Director: _____________________________________________Date: ______________

Joan Blackmon, Lubbock RSVP Director

Signature of RSVP Coordinator:___________________________________________Date:______________

Clay Ament, Lubbock RSVP Coordinator
